# Hissing of geese: caller identity encoded in a non-vocal acoustic signal

**DOI:** 10.7717/peerj.10197

**Published:** 2020-11-24

**Authors:** Richard Policht, Artur Kowalczyk, Ewa Łukaszewicz, Vlastimil Hart

**Affiliations:** 1 Department of Game Management and Wildlife Biology, Faculty of Forestry and Wood Sciences, Czech University of Life Sciences Prague, Praha, Czech Republic; 2 Division of Poultry Breeding, Institute of Animal Breeding, Wrocław University of Environmental and Life Sciences, Wroclaw, Poland

**Keywords:** Non-vocal, Communication, Vocal individuality, Non-syrinx vocalization, Recognition, Acoustic, Behavior, Anseriformes, Hiss-display, Bird

## Abstract

Non-vocal, or unvoiced, signals surprisingly have received very little attention until recently especially when compared to other acoustic signals. Some sounds made by terrestrial vertebrates are produced not only by the larynx but also by the syrinx. Furthermore, some birds are known to produce several types of non-syrinx sounds. Besides mechanical sounds produced by feathers, bills and/or wings, sounds can be also produced by constriction, anywhere along the pathway from the lungs to the lips or nostrils (in mammals), or to the bill (in birds), resulting in turbulent, aerodynamic sounds. These noises often emulate whispering, snorting or hissing. Even though hissing sounds have been studied in mammals and reptiles, only a few studies have analyzed hissing sounds in birds. Presently, only the hissing of small, nesting passerines as a defense against their respective predators have been studied. We studied hissing in domestic goose. This bird represents a ground nesting non-passerine bird which frequently produces hissing out of the nest in comparison to passerines producing hissing during nesting in holes e.g., parids. Compared to vocally produced alarm calls, almost nothing is known about how non-vocal hissing sounds potentially encode information about a caller’s identity. Therefore, we aimed to test whether non-vocal air expirations can encode an individual’s identity similar to those sounds generated by the syrinx or the larynx. We analyzed 217 hissing sounds from 22 individual geese. We calculated the Potential for Individual Coding (PIC) comparing the coefficient of variation both within and among individuals. In addition, we conducted a series of 15 a stepwise discriminant function analysis (DFA) models. All 16 acoustic variables showed a higher coefficient of variation among individuals. Twelve DFA models revealed 51.2–54.4% classification result (cross-validated output) and all 15 models showed 60.8–68.2% classification output based on conventional DFA in comparison to a 4.5% success rate when classification by chance. This indicates the stability of the DFA results even when using different combinations of variables. Our findings showed that an individual’s identity could be encoded with respect to the energy distribution at the beginning of a signal and the lowest frequencies. Body weight did not influence an individual’s sound expression. Recognition of hissing mates in dangerous situations could increase the probability of their surviving via a more efficient anti-predator response.

## Introduction

Vocalizations in terrestrial tetrapods are usually generated by vibrations in a specialized vocal organ, the syrinx or the larynx. Sublaryngeal/subsyringeal pressure from the lungs activates laryngeal vocal folds to generate a fundamental sound, which is then filtered by the upper vocal tract (Squire, 2009). The latter comprises the trachea, the pharynx and associated nasal and buccal cavities, with the adjunction of air sacs in some species (Favaro et al., 2015; Fitch & Hauser, 2002; Fletcher, 1993). This represents most studied systems, but sounds of terrestrial vertebrates are not only produced in the larynx or syrinx. Several avian species are known to produce non-syrinx sounds, which can be produced by instrumental feathers (Murray, Zeil & Magrath, 2017), beak drumming (Budka et al., 2018; Dodenhoff, Stark & Johnson, 2001), wings beating (Garcia et al., 2012; O’Neil, Charrier & Iwaniuk, 2018), the rattling of mandibles (Eda-Fujiwara et al., 2004), step dances (Ota, Gahr & Soma, 2017) or else, by using tools (Heinsohn et al., 2017).

Besides these emissions, sounds can be produced by constriction, anywhere along the pathway from the lungs to the lips or nostrils (in mammals), or to the bill (in birds), resulting in turbulent, aerodynamic sounds (Fitch & Hauser, 2002). As of late, non-vocal, or unvoiced, signals have surprisingly received very little attention in comparison to other acoustic signals (Budka et al., 2018; Stomp et al., 2018a). The production mechanism of hissing has been studied in the domestic goose (Brackenbury, 1978). The sound is produced during a long expiration, lasts for many seconds and is preceded by a deep inspiration. Air sac pressure and flow rate reach an intermediate level, somewhere between normal breathing and vocalization. By comparing maximum pressure and flow excursions during an inspiration and expiration, it has been shown that hissing is caused by a stream of expiratory air escaping from the constricted glottis (Brackenbury, 1978). In terrestrial animals such signals are used by reptiles and mammals: snakes (Aubret & Mangin, 2014), lizards (Labra et al., 2007), crocodiles (Vergne, Pritz & Mathevon, 2009), turtles (Fitch & Hauser, 2002), rhinos (Policht et al., 2008), horses (Stomp et al., 2018a; Stomp et al., 2018b), giraffes (Volodina et al., 2018), llamas (Fitch & Hauser, 2002), both house and feral cats (Yeon et al., 2011), tigers (Rose et al., 2018), cheetahs (Smirnova et al., 2016), sloths (Fitch & Hauser, 2002), dasyurids (Dorph & McDonald, 2017) and number of invertebrates as well, e.g., cockroaches (Hunsinger et al., 2018), beetles (Lewis & Cane, 1990), sphinx caterpillars (Dookie et al., 2017), Bombycoidea caterpillars (Bura, Kawahara & Yack, 2016), honeybees (Sarma et al., 2002), mantids (Hill, 2007). Even though hissing has been studied in mammals and reptiles, only few studies have been conducted in birds, mostly in the context of parental behavior in avian species nesting in cavities or burrows. Although hissing outside the nest is common in some ground-dwelling, non-passerines, like geese and swans, we are unaware of studies that tested hissing produced by adult birds that do not nest in cavities.

Geese and swans often protect their territories and have been known to violently chase away all intruders, birds or other animals alike, including approaching humans. Both offspring and adults can easily be caught by a predator on the ground. After producing a warning hiss, they usually attack with flapping wings and biting (Lorenz, 1963; Rosiński, 1986). The production of a sudden, intense sound during such an event can deter or surprise a potential predator, but there could be other functions, e.g., to attract conspecifics for pair or collective defense, or to warn other conspecifics. In any such case, hissing sounds may contain information about social structures (e.g., group or pair membership) or an individual’s identity.

Previous studies have shown the following functions of animal hissing: fear expression (Kinstler, 2009), to deter a predator (Broughton, 2005; Krams et al., 2014; Labra et al., 2007; Morley, 1953; Zub et al., 2017), acoustic mimicry (Aubret & Mangin, 2014), aggressive interactions between conspecifics (Policht et al., 2008; Rose et al., 2018; Smirnova et al., 2016) or during vigilance (Volodina et al., 2018). These sounds occur predominantly in dangerous situations, although it was also found to be a positive exhibition in horses (Stomp et al., 2018a; Stomp et al., 2018b).

Recognition of individuals during dangerous situations can be adaptive when signals provide additional important information e.g., relevancy of a threat or its urgency. For example, the calling of younger, inexperienced individuals can be less relevant than those from more experienced adults (Nakano et al., 2013; Sloan & Hare, 2008). Similarly, recognition of hissing mates in dangerous situations could increase the probability of their surviving via a more efficient anti-predator response. In comparison to vocally produced alarm calls, almost nothing is known whether non-vocal hissing sounds potentially encode information about the caller’s identity. Even though individual expressions within the vocalizations of birds have been intensively studied, the majority of studies have been devoted to the sounds produced by the syrinx. Such studies of non-syrinx vocalizations remain scarce, with little being known about their sound production mechanisms or their biological functions, including within and between-individual variation (Budka et al., 2018). The extent to which an individual’s variation occurs in turbulent sound like hissing has not, however, been tested. In this study, we tested the potential information contained in the hissing sounds of domestic geese, a non-passerine bird. We aimed to test the level of individual distinctiveness and the influence, if any, of sex and body weight.

## Materials & Methods

### Ethics statement

All applicable institutional, national and international guidelines for the care and use of animals were followed. The study has been conducted in accordance with the current laws in Poland. The university farm was responsible for goose husbandry, care and research manipulation in accordance with the regulation of Ministry of Agriculture and Rural Development (Dz. U. 2010 nr 116 poz. 778).

### Birds, recording and acoustic analysis

Observations were made on a parent flock of Bilgoraj geese kept in a deep litter, under controlled environmental conditions with free access to limited catwalks. Food and water were provided ad libitum to naturally mated birds. Geese were kept in one-parent flocks consisting of 230 individuals. Males formed harem flocks with approximately four females to each group. We did not record which males mated with which females. During testing, the specimens being tested were separated from their respective flock and were transferred to a separate room. Geese were recorded while a human approached the focal bird up to a distance of one meter. Hissing displays were defined as the action of a neck extending towards a person, stretched directly to the opponent with an opened bill, either moving or standing. We analyzed calls produced only at a close vicinity.

For recording we used a QTC50 microphone (Earthworks Inc. Milford, NH, USA, frequency response 3 Hz–50 kHz) and a ZOOM H5 digital audio recorder set to 44.1 kHz sampling rate and 16-bit sample size. After recording, each bird was weighed on an electronic balance (RADWAG WPT/R/6/15c2) with an accuracy up to 2.5 g and identified individually based on wing marks with a unique number code. After the procedure, tested individuals were returned to their flock.

**Figure 1:**
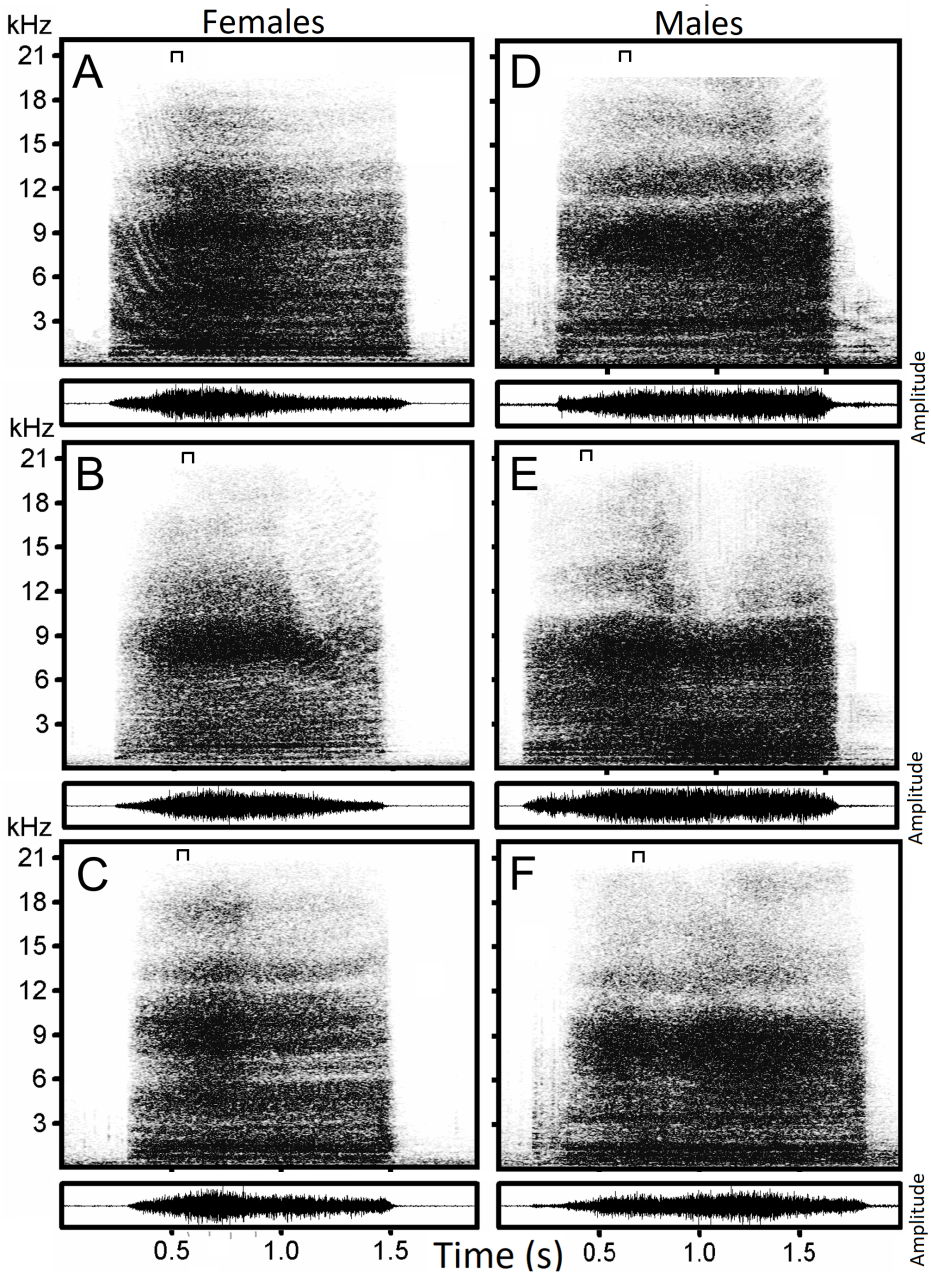
Examples of hissing calls recorded from six subjects. Each panel displays the spectrogram (top) and the amplitude modulation of the signal (below). Individual identity labeled according to the identity label used in DFA: (A) Female 4. (B) Female 5. (C) Female 13. (D) Male 18. (E) Male 19. (F) Male 20. The slice of sound energy representing the power spectrum is indicated by a symbol on top of each spectrogram.

We analyzed 217 hissing sounds (Fig. 1) from 22 individual geese (16 females and 6 males), produced within 5–15 min of separation from members of their respective flock. Recordings were analyzed using the Raven Pro Sound Analysis Software (Cornell Lab of Ornithology, New York, USA) from which spectrograms were generated using the following parameters: Hann window type with a 1,050 point window size, 50% overlap, 11.9 ms hop size, and 21 Hz grid spacing.

For the analysis, we visually inspected the quality of each spectrogram and selected calls with high signal-to-noise ratios that did not overlap with background noise. For the analysis we retained calls that did not differ in their root mean square amplitude (RMS). Prior to sound analysis, recordings were normalized (scaled from 0 to 1) to standardize loudness. Birds, recording and acoustic analysis Then we randomly selected the 10 best calls, or those with the highest quality, from each individual using a randomization function in IBM SPSS 20 (IBM Corp., Armonk, USA). One specimen produced only 7 calls, but was also included in the analysis.

We filtered out all background noise that was not overlapping with the frequency range of the hissing using a high pass filter on frequencies <130 Hz in Avisoft-SASLab Pro, version 5.2.13 software. We measured the following parameters (Table 1): Low frequency (LowF), Center frequency (CF), First quartile frequency (Q1F), Third quartile frequency (Q3F), First quartile time (Q1Time), Third quartile time (Q3Time), Time 95% (Time95), Time 5% (Time5), Call duration (Sample Length), Frequency 5% (F5), Frequency 95% (F95), Bandwidth 90% (BW90), Inter quartile range (IQR), Peak frequency (PF), Aggregate entropy (Agg Entropy) and Minimum entropy (Min entropy). For a more detailed description of these parameters, see Charif, Waack & Strickman (2010).

**Table 1:** Measured acoustical variables. Measurements based on the Raven Pro manual (Charif, Waack & Strickman, 2010).


**(LowF) Low frequency.**Minimum frequency of the signal. (Hz)
**(CF) Center frequency.**The frequency that divides a signal into two frequency intervals of equal energy. (Hz)
**(Q1F) First quartile frequency.**The frequency that divides the signal into two frequency intervals containing 25% and 75% of the energy. (Seconds)
**(Q3F) Third quartile frequency.**The frequency that divides the selection into two frequency intervals containing 75% and 25% of the energy. (Hz)
**(Q1T) First quartile time.**The point in time that divides the selection into two time intervals containing 25% and 75% of the energy. (Seconds)
**(Q3T) Third quartile time.**The point in time that divides the selection into two time intervals containing 75% and 25% of the energy. (Seconds)
**(T95) Time 95%.**The point in time that divides the signal into two time intervals containing 95% and 5% of the energy. (Seconds)
**(T5) Time 5%.**The point in time that divides the signal into two time intervals containing 5% and 95% of the energy. (Seconds)
**(Call duration) Sample length.** Signal duration based on sample length. (Samples)
**(F5) Frequency 5%.**The frequency that divides the signal into two frequency intervals containing 5% and 95% of the energy. (Hz)
**(F95) Frequency 95%.** The frequency that divides the selection into two frequency intervals containing 95% and 5% of the energy in the selection. (Hz)
**(BW90) Bandwidth 90%.**The difference between the 5% and 95% frequencies. (Hz)
**(IQR) Inter-quartile range.**The difference between the 1st and 3rd quartile frequencies. (Hz).
**(PeakF) Peak frequency.**Frequency of the maximum amplitude. (Hz)
(**AggE) Aggregate Entropy.** The aggregate entropy measures the disorder in a sound by analyzing the energy. Higher values correspond to greater disorder in the signal whereas a pure tone have zero entropy. It corresponds to the overall disorder in the sound.
**(MinE) Minimum Entropy.** This entropy is calculated by finding the entropy for each frame in the signal and then taking the minimum values.
The entropy formula: *S* = *PSD*(*f*, *t*)∕*sum*_*over*_*f*(*PSD*(*f*, *t*))∗*log*2(*PSD*(*f*, *t*)∕*sum*_*over*_*f*(*PSD*(*f*, *t*)))
The units are “bits” because we use the log base 2. Since the selection may consist of multiple spectrogram slices, Raven iterates over slices and to find the minimum and maximum entropy value with the frequency bounds of the selection. Note that most signal processing applications sum over frequency and time, where Raven sums over frequency instead.

### Statistical analysis

For each of the 16 sound features (Table 1), we performed Kruskal-Wallis tests with Bonferroni corrections on the non-transformed data to determine whether individual birds differed from one another. For each parameter, the Kruskal-Wallis test with Bonferroni correction showed significant differences among individuals (*p* < 0.001; Table 2).

**Table 2:** Descriptive statistics and Potential for individual coding. (DFA) variable included in DFA model. (SE) standard error of the mean. (Krusk–Wallis) Kruskal–Wallis test after Bonferroni correction. (**) *p* < 0.001. Mean CVw (within individual comparison, *n* = 22). CVa (between individual comparison, *n* = 217).

Variable	DFA	Mean	Min	Max	SE	Krusk–Wallis	Mean CVw	CVa	PIC
Frequency 5% (Hz)	X	821.1	280.0	3359.0	29.77	**	27.14	53.41	1.97
Time 5% (ms)		263.3	41.0	687.0	8.93	**	28.87	49.98	1.73
Min Entropy	X	5.9	4.0	8.0	0.06	**	12.05	15.17	1.26
Agg Entropy		8.4	6.0	9.0	0.04	**	5.98	7.80	1.30
Low frequency (Hz)		248.0	158.0	784.0	6.82	**	19.46	40.48	2.08
First quartile frequency (Hz)	X	1920.7	560.0	7795.0	91.13	**	40.01	69.89	1.75
First quartile time (ms)	X	263.6	41.0	687.0	8.93	**	28.83	49.92	1.73
Third quartile frequency (Hz)	X	6452.9	1378.0	9281.0	130.74	**	20.88	29.85	1.43
Third quartile time (ms)		264.1	42.0	687.0	8.93	**	28.76	49.83	1.73
Bandwidth 90% (Hz)	X	8702.4	3295.0	11908.0	76.45	**	9.74	12.94	1.33
Center frequency (Hz)		3900.4	775.0	8549.0	137.37	**	38.18	51.88	1.36
Call duration (samples)	X	64124.0	29892.0	133643.0	1212.51	**	15.87	27.85	1.75
Peak frequency (Hz)		2344.2	302.0	9044.0	164.37	**	65.11	103.29	1.59
Inter-quartile range (Hz)		4532.3	302.0	7235.0	108.98	**	26.88	35.42	1.32
Time 95% (ms)		264.3	42.0	687.0	8.93	**	28.74	49.79	1.73
Frequency 95% (Hz)		9223.4	4005.0	13286.0	83.85	**	9.22	12.97	1.41

As an index of inter-individual variation we calculated the Potential for Individual Coding (PIC), which compares the coefficient of variation both within and among individuals. The PIC ratio was computed for each acoustic parameter by dividing the CVbetween by the mean of the CVintra values obtained from each individual (Robisson et al. 1993).

In order to determine whether each hissing sound could be correctly classified, we performed a stepwise procedure of discriminant function analysis (DFA). The analysis selected predictors using the Wilks’ lambda criterion. *F* values were used as a criterion for entering or removing a parameter from a discrimination model (F-to enter = 3.84; F-to-remove = 2.71). We also used a leave-one-out cross-validation procedure for external validation using an IBM SPSS 20 (IBM Corp., Armonk, USA). The identity of the calling bird was used as a group identifier and the 16 acoustic parameters were used as discriminant variables. Normal distribution was tested (Kolmogorov–Smirnov test) and the data were transformed into the Box–Cox when necessary. Measured variables were normalized using *Z* score transformations (by subtracting the mean and dividing by the variable’s standard deviation) which avoided the false attribution of weights in relation to acoustic parameters measured in different units (IBM Corp., Armonk, USA).

## Results

**Figure 2:**
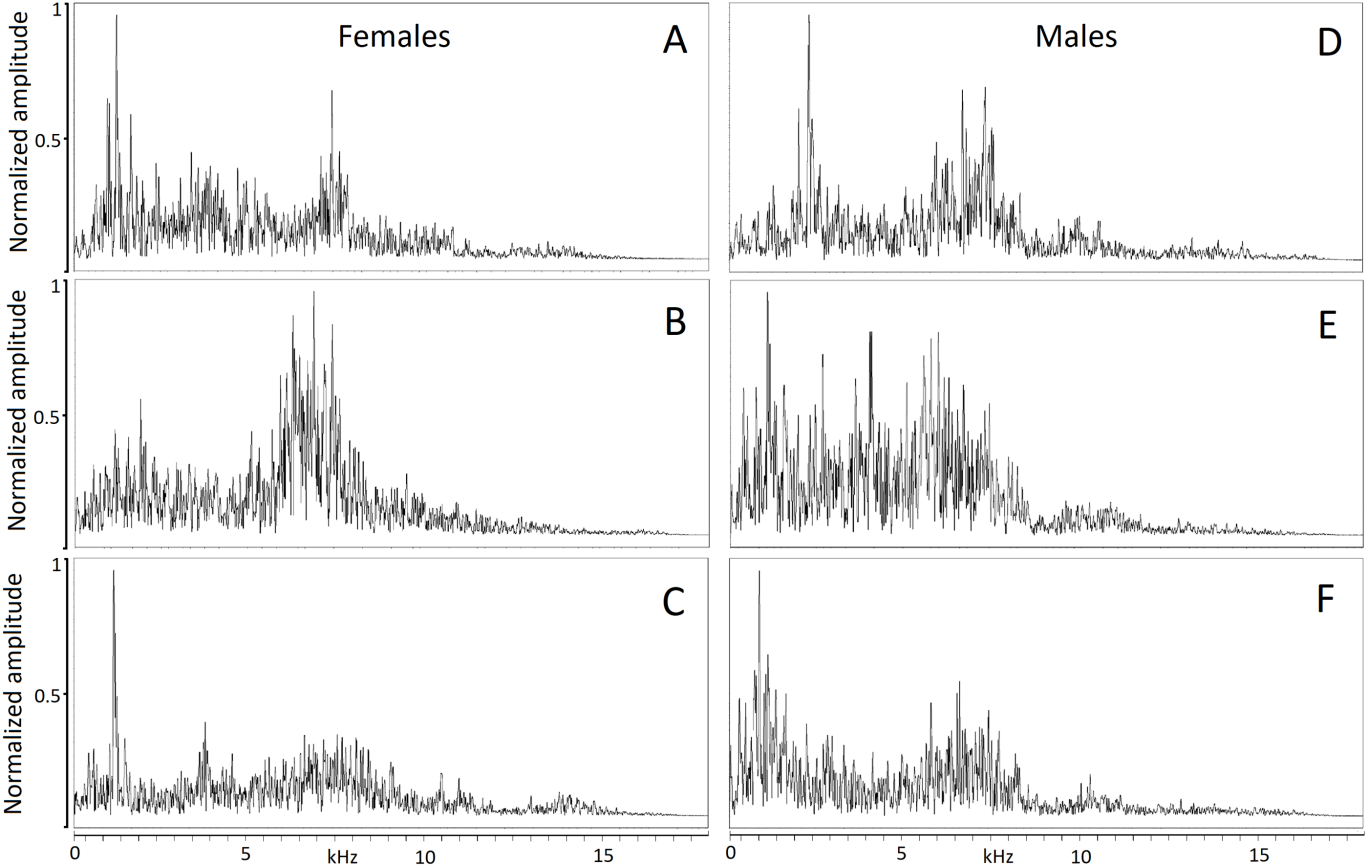
Powerspectrum of the hissing calls produced by six individuals showing individually distinct pattern. Individual identity labeled according to the identity label used in DFA: (A) Female 4. (B) Female 5. (C) Female 13. (D) Male 18. (E) Male 19. (F) Male 20. Power spectrum was taken from the 0.05 s interval of the first quartile time. This parameter mostly contributed to individual distinctiveness.

The hiss is a broadband and sustained sound combining a complex structure of stacked harmonics and overtones with a frequency modulation exhibiting one or more time-varying spectral peaks of energy. In some individuals, the hiss displays a sharp attack and ending (Figs. 2A, 2B, 2C) while in others, it begins or ends with a soft acoustic component (Figs. 2D, 2E, 2F). The pronounced spectral bands embedded in the call (e.g., Fig. 2B) may be formants resulting from the conformation of the vocal tract during expiration but we did not have anatomic data to support this.

**Figure 3:**
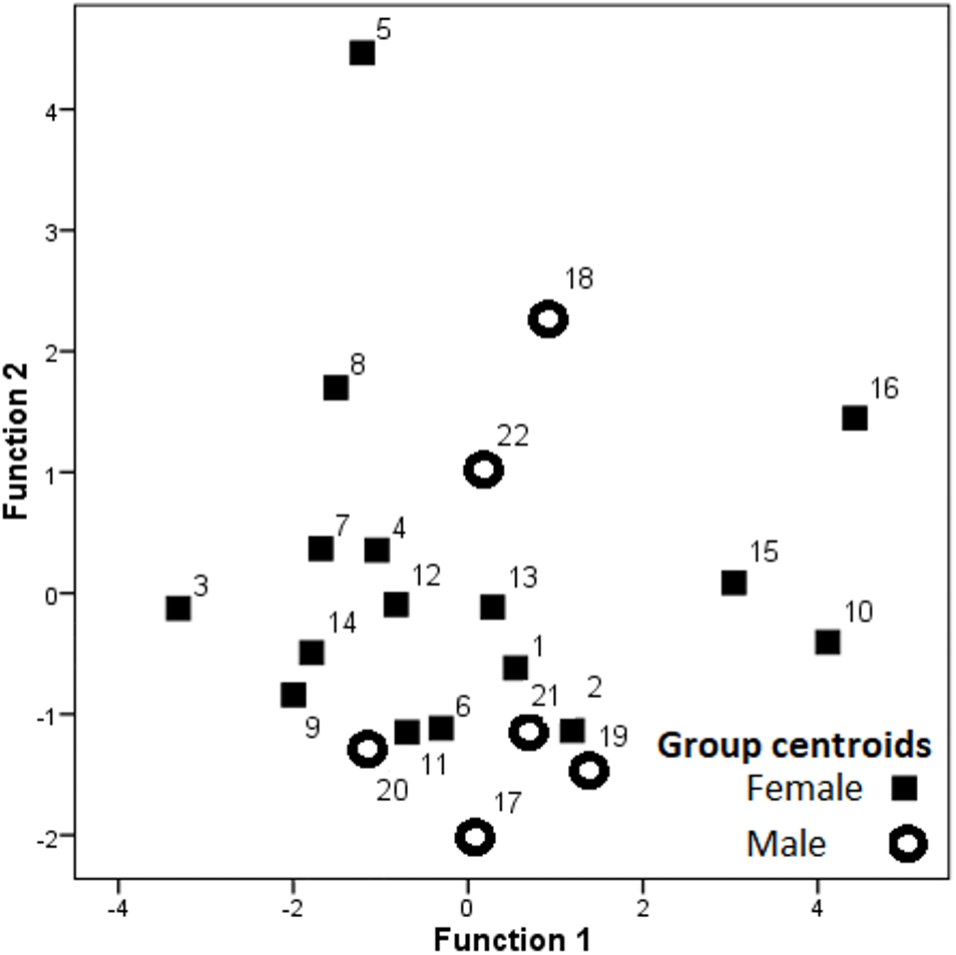
Dispersion of the group centroids on the two discriminant functions. Labels denote individual birds.

**Figure 4:**
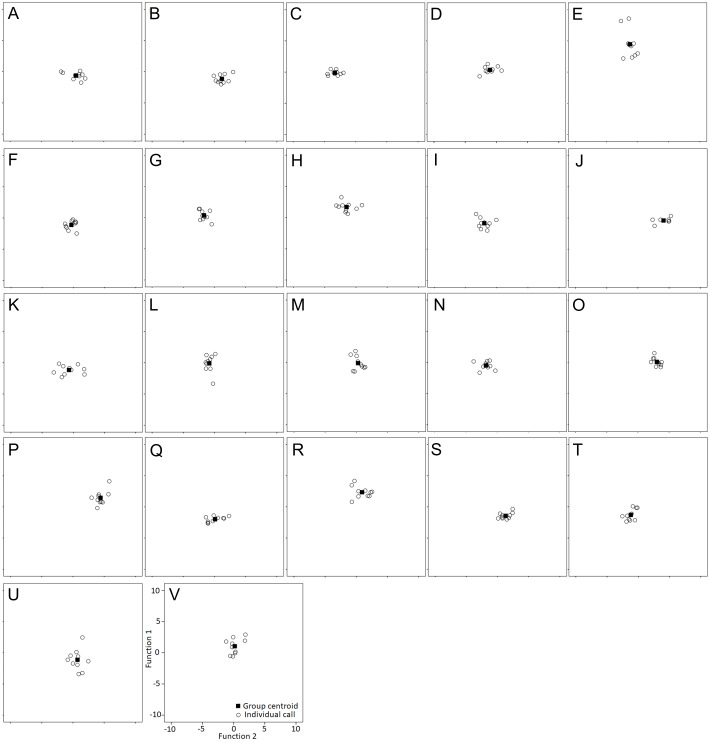
Individual scores are plotted with their respective centroid against the first two discriminant functions. Individual identity labeled according to the identity label used in DFA: (A–P) Female 1-16. (Q–V) Male 17–22.

Hissing calls reached a duration of 0.68–3.03 s (1.45 ±  0.03 s; mean ± SE). Peak frequency showed 302.0–9,044.0 Hz (2,344.2 ±  164.4), Inter-quartile ranged from 302.0–7,235.0 Hz (4,532.3 ± 109.0) and Low frequency at 158.0–784.0 Hz (248.0 ± 6.8), (Table 2). After Bonferroni correction, the Kruskal–Wallis test for each independent acoustic variable showed significant differences among individuals (*p* < 0.001) (Table 2). The DFA model contained seven significant discriminant functions: Q1T, Call duration, F5, Q3F, BW 90, MinE and Q1F (*p* < 0.001; Table 3). The PIC value was ≥1.26 for all 16 acoustic variables. This model (Wilks’ Lambda = 0.004; *P* < 0.001) included the first two discriminant functions, with Eigenvalues > 2 representing 59.7% of variation (Fig. 3). The first four functions had Eigenvalues > 1 and explained 87.8% of the variation (Table 3). The first discrimination function correlated with Q1T (*r* = 0.736), and the second discrimination function correlated with F5 and Q1F (r = 0.846 and 0.590, respectively). Classification results revealed that any random hissing sound could be assigned to the correct individual with an accuracy rate of 67.7%. A cross- validated result showed a success rate of 54.4% in comparison to a 4.5% success rate when classification by chance. Nine individuals reached the high classification accuracy (80–100%) (Table 4). The eigenvalues of individual sounds with their respective centroid are plotted against the first two discriminant functions (Figs. 3–4). In order to see the stability of this DFA result, we conducted a series of 15 DFA models (Table 5). Twelve DFA models revealed 51.2–54.4% classification result (cross-validated output) and all 15 models showed 60.8–68.2% classification output based on conventional DFA. This indicates the stability of the DFA results when using different combinations of variables. Such results show that signaler identity is encoded in a larger set of acoustic features. The influence of body weight was inconclusive as a factor with respect to any of the acoustic parameters and did not show a significant correlation (*r* <  − 0.34; *p* > 0.05).

**Table 3:** Discrimination functions of the DFA model. (Funct) DFA function. (Eigenval) eigenvalue, (Cum var, %) explained cumulative variance). (Wilks’ Lam) Wilks’ Lambda. (Sig) Significance. (**) *p* < 0.001.

Funct	Eigenval	Cum var (%)	Wilks’ Lam	Sig.	Mostly correlated variable
1	3.801	36.8	0.271	**	First quartile time (0.736)
2	2.364	59.7	0.091	**	Frequency 5% (0.846)
3	1.618	75.4	0.031	**	Call duration (0.693)
4	1.286	87.8	0.015	**	Third quartile frequency (0.798)
5	0.572	93.4	0.009	**	Bandwidth 90% (0.885)
6	0.449	97.7	0.006	**	Minimum Entropy (0.848)
7	0.234	100.0	0.004	**	First quartile frequency (0.442)

**Table 4:** Classification results. (ID) Individual identity. (Prior(%)) Prior probabilities of individuals. (DFA(%)) Percentage of correct classification.

ID	Sex	Nu calls	Prior(%)	DFA(%)
1	F	10	4.6	40
2	F	10	4.6	50
3	F	10	4.6	100
4	F	10	4.6	80
5	F	10	4.6	60
6	F	10	4.6	80
7	F	10	4.6	80
8	F	10	4.6	50
9	F	10	4.6	80
10	F	7	3.2	100
11	F	10	4.6	20
12	F	10	4.6	70
13	F	10	4.6	70
14	F	10	4.6	90
15	F	10	4.6	100
16	F	10	4.6	80
17	M	10	4.6	70
18	M	10	4.6	70
19	M	10	4.6	60
20	M	10	4.6	40
21	M	10	4.6	50
22	M	10	4.6	60

The sample size of males studied was too small to make an accurate assessment about any potential differences between sexes (*p* > 0.25, Mann–Whitney *U* Test).

## Discussion

The present study demonstrates that the hissing of geese, a non-vocal signal elicited in a standard experimental setup, encodes signaler identity. Our discriminant analysis found individuality embedded in specific acoustic features, which is also typical for broadband vocal signals of many animals. In the best DFA model two acoustic parameters among the sixteen that we analyzed proved to be important in separating individuals from one another: the beginning of a hiss (Q1T) and the lowest frequency (F5).

For the other 14 DFA models, in addition to Q1T, the first discrimination function mostly correlated with two other time parameters (T5 and Q3T). Three other frequency parameters (Q1F, CF and Q3F) were most often correlated with the second discrimination function. Such results show that signaler identity is encoded in a larger set of acoustic features.

In addition to F5 of the most explanatory model, three other frequency parameters were most often correlated with the second discrimination function (F25%, F50 and F75, Q1F, CF and Q3F, respectively). Such results show that identity of the signaler is encoded in a larger set of acoustic features.

**Table 5:** DFA models.

DFA	Nu of var	Conv class	Valid class	Variables (ordered by importance, starting with the most explanatory variable)
1	7	67.7	54.4	Q1T; Duration; F5; Q3F; BW90; Min Entr; Q1F
2	8	67.3	54.4	T5; Duration; Q3F; IQR; Q1F; BW90; Min Entr; Low F
3	6	65.0	53.0	Q3T; Duration; Q3F; Q1F; BW90; Min Entr
4	7	67.3	54.4	Q3T; Duration; Q3F; Q1F; BW90; Min Entr; Low F
5	7	67.7	53.9	T5; Duration; F5; Q3F; BW90; Min Entr; Q1F
6	8	68.2	53.9	T5; Duration; F5; IQR; Min Entr; Q3F; Q1F; BW90
7	8	66.4	52.1	T5; Duration; Q3F; IQR; Q1F; BW90; Min Entr;F95
8	7	63.6	51.2	T95; Duration; F5; IQR; Q3F; BW90; Centr F
9	7	63.6	51.2	Q3T; Duration; F5; IQR; Q3F; BW90; Centr F
10	7	66.4	51.2	T5; Duration; F5; IQR; Min Entr; Q3F; BW90
11	8	65.0	51.2	T5; Duration; Q3F; IQR; Agg Entr; Min Entr; BW90; F95
12	7	62.7	53.9	T95; Duration; Q3F; Centr F; Min Entr; Pak F; F 95
13	7	60.8	48.4	T5; Duration; Q3F; IQR; Agg Entr; Min Entr; BW 90
14	7	60.8	48.4	T95; Duration; Q3F; IQR; Agg Entr; Min Entr; BW90
15	6	62.7	47.9	T5; Duration; Q3F; BW90; F95; Min Entr

Inter-individual variation of this non-vocal sound produced by constriction of the glottis during exhalation could result from differences in vocal tract anatomy and/or individual morphology (e.g., body size, body weight) in accordance with general principles of allometry. However, none of the acoustic parameters we analyzed correlated with body weight. Although we cannot exclude the influence of other factors, such as the length or the shape of the trachea, individual distinctiveness of this unvoiced call does not appear to be driven by morphological differences. The songs of some passerines have been found to be constrained by the condition of a specimen’s immune system (Gil & Gahr, 2002), parasite load (Garamszegi 2005) or MHC profile (Garamszegi et al., 2018). Similarly, distress calls have been found influenced by an individual’s health status (Laiolo et al., 2007; Laiolo et al., 2004).

Alternatively, the inter-individual variation found in hissing geese could result from a selective process favoring conspecific recognition. To test this hypothesis, it would be useful to collect data from wild geese living in pairs instead of domestic geese bred in flocks. This would give us an opportunity to compare within-pair and between-pair variation during performance of a joint hissing display. Subsequently, we could investigate, using playback, whether subjects are more responsive to the hissing of his/her mate relative to a stranger’s.

The hissing display of geese could be considered as a component of parental investment. In contrast to small passerines, in which hissing is produced only by females during incubation or brood rearing, male geese often produce hissing jointly with their mate. However, male geese are not always present to warn and defend their mate against a predator (Perrins, 1979). Like other displays (e.g., greeting ceremony), the hissing of geese is often performed in synchrony by both mates. During the display, females may exploit the variations in expressions to test the ability of males to potentially invest in a family unit and protect it from danger.

The hissing of geese may be energetically costly as the sound is preceded by a deep inhalation and is released during a prolonged exhalation, which can last many seconds (Brackenbury, 1978). Its emission could be constrained by an individual’s condition. Females could evaluate the fitness of their mate based on the quality of his hissing. Males in better physical condition produce longer or better quality hissing sounds although, in our study, we did not find any correlation with body weight.

Our results provide evidence that the hissing of geese may advertise an individual’s identity to a potential receiver whereas sex and body weight were inconclusive with respect to this call. Future studies could test the influence of additional individual qualities (e.g., health condition, social status, etc.). This would expand our knowledge on how non-vocal signals could potentially encode such information.

We studied hissing in a domesticated bird bred for meat production, but geese are known to retain many attributes of their wild counterparts (Kozak, 2019; Łukaszewicz et al. 2019). Furthermore, hissing is common in many anserine birds (del Hoyo et al. 1994). Although there are notable behavioral differences between wild and domestic animals, many similarities still persist (Jensen 2002). Each behavior is partly under the control of genetic mechanisms, which have been adapted and designed over thousands of generations of evolution in nature and domestication has altered them only slightly (Jensen 2002).

Compared with pair-living wild geese, we do not think that the farming environment in which subjects were kept in one parent flock could selectively act against vocal individuality. Individual vocal recognition has been frequently documented in birds living in flocks. For example, colonial birds provide a robust model for research on individual acoustic recognition (e.g., penguins: Aubin & Jouventin 2002; Aubin, Jouventin & Hildebrand, 2000; Jouventin, Aubin & Lengagne, 1999; gulls: Aubin et al. 2007; Beer 1969; skuas: Charrier et al. 2001; swallows:  Beecher & Beecher 1983; Beecher, Beecher & Hahn, 1981; parrots: Berg et al. 2011; Wanker & Fischer 2001). Besides, wild geese also spend part of their life cycles gathering in flocks (Cramp & Simmons, 1980; Del Hoyo et al. 1994). We do not think our results might indicate pair-specific rather than individual characteristics because we found significantly higher inter-individual variations in comparison to intra-individual variations. Besides, the flock of geese we studied consisted of harems with approximately four females to one male. We would expect pair-specific characteristics to prevail in duetting birds, as has been found in cranes that have both individually specific calls (Klenova et al. 2009b; Klenova et al. 2008b) and pair specific calls produced either in unison or as a duet (Klenova, Volodin & Volodina, 2008a; Klenova et al. 2009a).

The study also raises questions about the function of this signal, i.e., what responses such hissing behavior evokes in the receivers. Playback experiments could potentially reveal whether family members or flock members respond more strongly than other individuals. However, we do have observations showing that hissing produced by an “attacked” individual attracts companions, resulting in “collective” hissing display. Recognition of hissing sounds produced by a threatened individual may trigger affective behaviors such as collective defense (pair, family, flock), which is typical for geese and swans.

## Conclusions

This research is the first providing a description of the acoustic parameters of geese hissing.

We demonstrate that non-syrinx hissing sounds of geese vary between individuals more frequently than expected. Body weight proved to be inconclusive as an influential factor. Previous research of hissing sounds in birds have mainly studied hole-nesting birds in which this sound was produced by females in their respective nesting sites (Broughton, 2005; Morley, 1953). In contrast, our study deals with hissing produced by both females and males. Besides direct anti-predatory functions, hissing sounds certainly attract pairings/ mate-matching or warn other conspecifics (e.g., family or flock members). The results indicate that this non-vocal sound can encode individually specific information in a similar way as the more frequently studied vocalizations produced by the syrinx.

##  Supplemental Information

10.7717/peerj.10197/supp-1 Supplemental Information 1 Measured data from acoustical analysis (ID) Identity corresponding to DFA results. (Sex) Sex of tested individual. (Weight) Body weight. (F5) Frequency 5%. (MinE) Minimum Entropy. (AggE) Aggregate Entropy. (LowF) Low frequency. (Q1F) First quartile frequency. (Q1Time) First quartile time. (Q3F) Third quartile frequency. (BW90) Bandwidth 90%. (CentF) Center frequency. (Call dur) Sample Length. (PeakF) Peak frequency. (IQR) Inter- quartile range. (T95) Time 95%. (T5) Time 5%. (Q3T) Third quartile time. (F95) Frequency 95%.

## Additional Information and Declarations

### Data Availability

The following information was supplied regarding data availability: The raw data are available in the Supplemental Files.
