# Supplementary material for: Hissing of geese: caller identity encoded in a non-vocal acoustic signal
Source: PeerJ. 2020 Nov 24;8:e10197. doi: 10.7717/peerj.10197 (PMC7694559; doi:10.7717/peerj.10197)
Supplement: Supplemental Information 1 — (ID) Identity corresponding to DFA results. (Sex) Sex of tested individual. (Weight) Body weight. (F5) Frequency 5%. (MinE) Minimum Entropy. (AggE) Aggregate Entropy. (LowF) Low frequency. (Q1F) First quartile frequency. (Q1Time) First quartile time. (Q3F) Third quartile frequency. (BW90) Bandwidth 90%. (CentF) Center frequency. (Call dur) Sample Length. (PeakF) Peak frequency. (IQR) Inter- quartile range. (T95) Time 95%. (T5) Time 5%. (Q3T) Third quartile time. (F95) Frequency 95%. [file peerj-08-10197-s001.docx]

| ID | Sex | Weight | F5 | MinE | AggE | LowF | Q1F | Q1T | Q3F | BW90 | CentF | Call dur | PeakF | IQR | T95 | T5 | Q3T | F95 |
| --- | --- | --- | --- | --- | --- | --- | --- | --- | --- | --- | --- | --- | --- | --- | --- | --- | --- | --- |
| 14 | F | 5,33 | 581 | 4 | 7 | 510 | 646 | 237 | 1378 | 7817 | 775 | 79379 | 625 | 732 | 238 | 236 | 237 | 8398 |
| 14 | F | 5,33 | 560 | 4 | 7 | 473 | 646 | 257 | 4113 | 7795 | 1012 | 72835 | 581 | 3467 | 258 | 257 | 258 | 8355 |
| 14 | F | 5,33 | 452 | 4 | 8 | 218 | 668 | 263 | 5017 | 8786 | 1766 | 81522 | 581 | 4350 | 264 | 263 | 263 | 9238 |
| 14 | F | 5,33 | 452 | 4 | 7 | 218 | 646 | 280 | 4457 | 8527 | 818 | 96890 | 711 | 3811 | 281 | 279 | 280 | 8979 |
| 14 | F | 5,33 | 517 | 5 | 8 | 236 | 1034 | 297 | 5039 | 8398 | 2606 | 92547 | 603 | 4005 | 298 | 297 | 298 | 8915 |
| 14 | F | 5,33 | 388 | 5 | 8 | 254 | 775 | 315 | 5319 | 8656 | 2649 | 76641 | 668 | 4544 | 316 | 315 | 316 | 9044 |
| 14 | F | 5,33 | 280 | 5 | 8 | 218 | 625 | 319 | 4307 | 8656 | 1184 | 120278 | 323 | 3682 | 320 | 318 | 320 | 8936 |
| 14 | F | 5,33 | 646 | 5 | 8 | 225 | 1314 | 328 | 5749 | 8463 | 2821 | 90543 | 1357 | 4436 | 329 | 327 | 328 | 9109 |
| 14 | F | 5,33 | 581 | 4 | 8 | 519 | 668 | 343 | 3941 | 7881 | 1227 | 133643 | 646 | 3273 | 345 | 343 | 345 | 8463 |
| 14 | F | 5,33 | 581 | 4 | 7 | 519 | 668 | 348 | 3725 | 8527 | 861 | 98895 | 732 | 3058 | 349 | 347 | 348 | 9109 |
| 3 | F | 4,30 | 689 | 5 | 7 | 261 | 1141 | 69 | 2821 | 8075 | 1378 | 58232 | 1184 | 1680 | 69 | 68 | 69 | 8764 |
| 3 | F | 4,30 | 711 | 5 | 6 | 236 | 1163 | 82 | 1464 | 3295 | 1292 | 91163 | 1206 | 302 | 83 | 82 | 83 | 4005 |
| 3 | F | 4,30 | 625 | 5 | 8 | 251 | 1120 | 91 | 2972 | 8656 | 1400 | 79517 | 1120 | 1852 | 92 | 90 | 91 | 9281 |
| 3 | F | 4,30 | 754 | 5 | 7 | 458 | 1034 | 100 | 2692 | 7774 | 1184 | 82730 | 1012 | 1658 | 101 | 100 | 101 | 8527 |
| 3 | F | 4,30 | 711 | 4 | 7 | 220 | 1055 | 118 | 2713 | 7601 | 1184 | 88352 | 1077 | 1658 | 119 | 118 | 119 | 8312 |
| 3 | F | 4,30 | 689 | 5 | 7 | 264 | 1120 | 143 | 2735 | 7903 | 1443 | 83534 | 1421 | 1615 | 144 | 143 | 144 | 8592 |
| 3 | F | 4,30 | 754 | 5 | 8 | 554 | 1292 | 165 | 3122 | 7881 | 2067 | 87951 | 711 | 1830 | 166 | 165 | 166 | 8635 |
| 3 | F | 4,30 | 689 | 4 | 7 | 581 | 797 | 169 | 2390 | 3747 | 1206 | 107228 | 797 | 1594 | 170 | 168 | 169 | 4436 |
| 3 | F | 4,30 | 668 | 5 | 8 | 229 | 1077 | 175 | 4544 | 8829 | 1378 | 73894 | 1120 | 3467 | 176 | 175 | 176 | 9496 |
| 3 | F | 4,30 | 431 | 5 | 8 | 264 | 1227 | 195 | 4264 | 8786 | 1400 | 91565 | 1227 | 3036 | 196 | 194 | 195 | 9216 |
| 6 | F | 4,68 | 581 | 5 | 8 | 174 | 1120 | 41 | 3467 | 8355 | 1400 | 35486 | 1378 | 2347 | 42 | 41 | 42 | 8936 |
| 6 | F | 4,68 | 323 | 5 | 8 | 224 | 840 | 72 | 4134 | 9173 | 1120 | 35794 | 1055 | 3295 | 73 | 72 | 73 | 9496 |
| 6 | F | 4,68 | 646 | 6 | 8 | 261 | 1163 | 77 | 4780 | 8398 | 1550 | 47520 | 1163 | 3618 | 78 | 77 | 77 | 9044 |
| 6 | F | 4,68 | 302 | 5 | 7 | 212 | 861 | 83 | 1443 | 9561 | 1034 | 41658 | 991 | 581 | 83 | 83 | 83 | 9862 |
| 6 | F | 4,68 | 625 | 5 | 8 | 187 | 1357 | 148 | 4651 | 8786 | 1938 | 35486 | 1400 | 3295 | 148 | 148 | 148 | 9410 |
| 6 | F | 4,68 | 345 | 7 | 8 | 212 | 1572 | 160 | 5986 | 9130 | 3768 | 41040 | 3811 | 4414 | 160 | 159 | 160 | 9475 |
| 6 | F | 4,68 | 625 | 5 | 8 | 212 | 1120 | 189 | 3811 | 7623 | 1507 | 46595 | 1120 | 2692 | 190 | 189 | 190 | 8247 |
| 6 | F | 4,68 | 366 | 4 | 6 | 212 | 1077 | 197 | 1400 | 5792 | 1206 | 31166 | 1378 | 323 | 197 | 197 | 197 | 6159 |
| 6 | F | 4,68 | 560 | 6 | 9 | 212 | 2821 | 224 | 7946 | 9862 | 4113 | 50298 | 3381 | 5125 | 225 | 224 | 225 | 10422 |
| 6 | F | 4,68 | 517 | 5 | 7 | 199 | 1012 | 235 | 3295 | 7580 | 1163 | 44744 | 1055 | 2283 | 235 | 235 | 235 | 8097 |
| 2 | F | 4,44 | 625 | 7 | 9 | 194 | 2842 | 261 | 8398 | 11908 | 5060 | 45170 | 3230 | 5556 | 261 | 261 | 261 | 12532 |
| 2 | F | 4,44 | 366 | 6 | 9 | 194 | 1012 | 264 | 7730 | 11456 | 4113 | 47367 | 646 | 6718 | 265 | 264 | 265 | 11822 |
| 2 | F | 4,44 | 560 | 6 | 9 | 202 | 3187 | 282 | 8290 | 9668 | 6697 | 52494 | 603 | 5103 | 282 | 281 | 282 | 10228 |
| 2 | F | 4,44 | 517 | 7 | 9 | 194 | 1335 | 288 | 7687 | 9324 | 4500 | 60796 | 668 | 6352 | 288 | 287 | 288 | 9841 |
| 2 | F | 4,44 | 409 | 6 | 9 | 202 | 1077 | 294 | 7644 | 10358 | 4436 | 65923 | 646 | 6568 | 294 | 294 | 294 | 10767 |
| 2 | F | 4,44 | 345 | 5 | 9 | 194 | 1163 | 302 | 7644 | 9302 | 4242 | 65679 | 646 | 6482 | 303 | 302 | 303 | 9647 |
| 2 | F | 4,44 | 452 | 5 | 9 | 176 | 1314 | 313 | 8506 | 10724 | 4931 | 74957 | 668 | 7192 | 314 | 313 | 314 | 11176 |
| 2 | F | 4,44 | 323 | 5 | 9 | 211 | 1098 | 324 | 7946 | 9733 | 4522 | 59087 | 1012 | 6848 | 325 | 324 | 324 | 10056 |
| 2 | F | 4,44 | 732 | 7 | 9 | 202 | 2885 | 363 | 7558 | 9948 | 5168 | 39798 | 3036 | 4673 | 363 | 363 | 363 | 10681 |
| 2 | F | 4,44 | 366 | 6 | 8 | 218 | 904 | 383 | 4156 | 8204 | 1507 | 51518 | 1012 | 3252 | 383 | 382 | 383 | 8570 |
| 7 | F | 5,49 | 1034 | 6 | 9 | 264 | 2907 | 93 | 8204 | 8936 | 5943 | 61408 | 2972 | 5297 | 93 | 92 | 93 | 9970 |
| 7 | F | 5,49 | 1184 | 5 | 9 | 282 | 2821 | 97 | 8010 | 8333 | 6589 | 64940 | 7364 | 5190 | 98 | 97 | 98 | 9518 |
| 7 | F | 5,49 | 1120 | 5 | 9 | 264 | 2907 | 110 | 7644 | 8549 | 6611 | 65212 | 7149 | 4737 | 111 | 110 | 110 | 9668 |
| 7 | F | 5,49 | 1163 | 6 | 9 | 264 | 3036 | 115 | 7774 | 8613 | 6740 | 65756 | 6740 | 4737 | 116 | 115 | 115 | 9776 |
| 7 | F | 5,49 | 775 | 5 | 8 | 246 | 1572 | 120 | 7687 | 8549 | 6266 | 69016 | 1184 | 6115 | 121 | 120 | 121 | 9324 |
| 7 | F | 5,49 | 861 | 6 | 8 | 261 | 2433 | 203 | 7537 | 8635 | 3208 | 77711 | 2972 | 5103 | 204 | 203 | 204 | 9496 |
| 7 | F | 5,49 | 711 | 4 | 8 | 246 | 1227 | 216 | 7580 | 8635 | 3661 | 65484 | 711 | 6352 | 216 | 215 | 216 | 9345 |
| 7 | F | 5,49 | 668 | 6 | 9 | 224 | 2993 | 230 | 8032 | 9130 | 6675 | 86949 | 7903 | 5039 | 231 | 230 | 231 | 9798 |
| 7 | F | 5,49 | 1077 | 5 | 8 | 249 | 1421 | 287 | 7515 | 8161 | 4285 | 76352 | 1443 | 6094 | 288 | 287 | 288 | 9238 |
| 7 | F | 5,49 | 495 | 5 | 8 | 286 | 926 | 311 | 6654 | 8484 | 2735 | 78254 | 904 | 5728 | 312 | 310 | 311 | 8979 |
| 13 | F | 4,24 | 1400 | 7 | 9 | 229 | 4910 | 139 | 8786 | 8829 | 7623 | 58819 | 7817 | 3876 | 140 | 139 | 140 | 10228 |
| 13 | F | 4,24 | 560 | 6 | 9 | 229 | 1357 | 203 | 8333 | 10271 | 4091 | 65291 | 1357 | 6977 | 204 | 203 | 203 | 10831 |
| 13 | F | 4,24 | 538 | 6 | 8 | 246 | 1055 | 224 | 6826 | 9625 | 1615 | 64910 | 1077 | 5771 | 225 | 224 | 225 | 10164 |
| 13 | F | 4,24 | 689 | 5 | 8 | 264 | 1077 | 269 | 8247 | 9604 | 2476 | 49301 | 1077 | 7171 | 270 | 269 | 270 | 10293 |
| 13 | F | 4,24 | 732 | 6 | 9 | 282 | 1271 | 274 | 8290 | 9798 | 4177 | 49873 | 1012 | 7020 | 275 | 274 | 275 | 10530 |
| 13 | F | 4,24 | 646 | 5 | 8 | 299 | 1012 | 293 | 6632 | 9302 | 1443 | 55202 | 1012 | 5620 | 293 | 293 | 293 | 9948 |
| 13 | F | 4,24 | 969 | 5 | 9 | 473 | 1378 | 299 | 8269 | 10293 | 3510 | 68717 | 1012 | 6891 | 300 | 299 | 300 | 11262 |
| 13 | F | 4,24 | 797 | 6 | 8 | 224 | 1034 | 308 | 8075 | 9625 | 1960 | 55012 | 1034 | 7041 | 309 | 308 | 309 | 10422 |
| 13 | F | 4,24 | 1206 | 6 | 9 | 224 | 3036 | 315 | 8979 | 10035 | 7860 | 77473 | 8592 | 5943 | 316 | 315 | 316 | 11240 |
| 13 | F | 4,24 | 926 | 6 | 8 | 224 | 1378 | 329 | 7817 | 8936 | 3165 | 75760 | 1378 | 6438 | 330 | 328 | 329 | 9862 |
| 5 | F | 3,78 | 3208 | 6 | 8 | 229 | 7687 | 133 | 9152 | 8118 | 8549 | 79292 | 8893 | 1464 | 133 | 132 | 133 | 11327 |
| 5 | F | 3,78 | 1421 | 6 | 9 | 246 | 4221 | 169 | 8915 | 9367 | 8032 | 82529 | 8807 | 4694 | 170 | 169 | 170 | 10788 |
| 5 | F | 3,78 | 3359 | 6 | 8 | 229 | 7795 | 184 | 9109 | 6826 | 8549 | 67502 | 9044 | 1314 | 184 | 183 | 184 | 10185 |
| 5 | F | 3,78 | 2024 | 6 | 8 | 246 | 6826 | 197 | 8678 | 8204 | 8053 | 69814 | 8226 | 1852 | 198 | 197 | 198 | 10228 |
| 5 | F | 3,78 | 1917 | 6 | 8 | 282 | 6718 | 204 | 8678 | 8721 | 8053 | 66115 | 8441 | 1960 | 205 | 204 | 205 | 10637 |
| 5 | F | 3,78 | 1378 | 5 | 9 | 264 | 3984 | 219 | 8484 | 8786 | 7687 | 54325 | 7817 | 4500 | 220 | 219 | 220 | 10164 |
| 5 | F | 3,78 | 2046 | 6 | 8 | 229 | 7214 | 225 | 8656 | 8183 | 8075 | 62879 | 8441 | 1443 | 225 | 224 | 225 | 10228 |
| 5 | F | 3,78 | 1981 | 6 | 8 | 246 | 7278 | 232 | 8893 | 9001 | 8183 | 79755 | 8075 | 1615 | 232 | 231 | 232 | 10982 |
| 5 | F | 3,78 | 1421 | 6 | 9 | 246 | 5146 | 239 | 8872 | 8678 | 7967 | 71433 | 8570 | 3725 | 240 | 239 | 240 | 10099 |
| 5 | F | 3,78 | 1400 | 5 | 9 | 246 | 3984 | 245 | 8592 | 9475 | 7429 | 52939 | 7881 | 4608 | 246 | 245 | 246 | 10874 |
| 4 | F | 4,12 | 560 | 5 | 8 | 212 | 1120 | 52 | 4931 | 9927 | 1486 | 62640 | 1184 | 3811 | 53 | 52 | 53 | 10487 |
| 4 | F | 4,12 | 1034 | 7 | 9 | 249 | 3575 | 74 | 8097 | 9712 | 5276 | 55639 | 4479 | 4522 | 75 | 74 | 75 | 10745 |
| 4 | F | 4,12 | 1206 | 7 | 9 | 336 | 2455 | 97 | 8656 | 9604 | 5448 | 67062 | 1529 | 6202 | 98 | 96 | 97 | 10810 |
| 4 | F | 4,12 | 1184 | 6 | 9 | 236 | 2433 | 112 | 8807 | 9776 | 5190 | 66325 | 1550 | 6374 | 113 | 112 | 113 | 10960 |
| 4 | F | 4,12 | 1227 | 7 | 9 | 212 | 1917 | 123 | 7881 | 9561 | 5319 | 62272 | 1572 | 5965 | 123 | 122 | 123 | 10788 |
| 4 | F | 4,12 | 1184 | 7 | 9 | 187 | 2864 | 172 | 8398 | 9130 | 5749 | 62640 | 1486 | 5534 | 172 | 172 | 172 | 10314 |
| 4 | F | 4,12 | 818 | 6 | 9 | 187 | 2024 | 192 | 7494 | 10013 | 4759 | 63745 | 1249 | 5469 | 193 | 192 | 193 | 10831 |
| 4 | F | 4,12 | 1098 | 6 | 8 | 236 | 1658 | 254 | 5943 | 8915 | 2929 | 84748 | 2885 | 4285 | 255 | 254 | 255 | 10013 |
| 4 | F | 4,12 | 861 | 6 | 9 | 274 | 3790 | 273 | 7860 | 9798 | 4802 | 60429 | 4393 | 4070 | 273 | 272 | 273 | 10659 |
| 4 | F | 4,12 | 689 | 5 | 9 | 236 | 1594 | 275 | 7709 | 10142 | 4091 | 49006 | 1206 | 6115 | 276 | 275 | 275 | 10831 |
| 8 | F | 3,90 | 1400 | 6 | 9 | 476 | 2326 | 109 | 6482 | 8484 | 4651 | 83925 | 6072 | 4156 | 110 | 108 | 110 | 9884 |
| 8 | F | 3,90 | 1034 | 6 | 9 | 398 | 1637 | 134 | 6482 | 9647 | 3230 | 61114 | 1443 | 4845 | 134 | 133 | 134 | 10681 |
| 8 | F | 3,90 | 1357 | 7 | 9 | 405 | 2110 | 139 | 6309 | 8484 | 4350 | 70814 | 2541 | 4199 | 140 | 139 | 140 | 9841 |
| 8 | F | 3,90 | 1314 | 7 | 9 | 548 | 1895 | 153 | 6417 | 8333 | 4027 | 75317 | 1615 | 4522 | 154 | 153 | 154 | 9647 |
| 8 | F | 3,90 | 1421 | 6 | 8 | 722 | 2110 | 163 | 5642 | 5642 | 4070 | 86077 | 6482 | 3531 | 164 | 162 | 163 | 7063 |
| 8 | F | 3,90 | 1443 | 5 | 9 | 498 | 2175 | 225 | 8376 | 11025 | 5060 | 92103 | 1550 | 6202 | 225 | 224 | 225 | 12468 |
| 8 | F | 3,90 | 1034 | 5 | 9 | 498 | 1637 | 227 | 6115 | 10250 | 2778 | 87368 | 1594 | 4479 | 228 | 227 | 228 | 11283 |
| 8 | F | 3,90 | 1141 | 6 | 9 | 224 | 2821 | 389 | 8635 | 10013 | 5663 | 109318 | 6051 | 5814 | 390 | 388 | 390 | 11154 |
| 8 | F | 3,90 | 1098 | 7 | 9 | 249 | 2692 | 393 | 8656 | 10874 | 5469 | 96406 | 2842 | 5965 | 394 | 392 | 394 | 11973 |
| 8 | F | 3,90 | 1184 | 7 | 9 | 224 | 2885 | 404 | 7020 | 9948 | 5211 | 80912 | 5857 | 4134 | 405 | 403 | 405 | 11133 |
| 9 | F | 5,19 | 991 | 6 | 9 | 199 | 3704 | 115 | 7730 | 8656 | 6266 | 87018 | 1012 | 4027 | 116 | 115 | 116 | 9647 |
| 9 | F | 5,19 | 969 | 7 | 9 | 274 | 3747 | 131 | 7709 | 9044 | 6761 | 85498 | 7537 | 3962 | 131 | 130 | 131 | 10013 |
| 9 | F | 5,19 | 711 | 7 | 9 | 224 | 2003 | 143 | 7709 | 10659 | 6245 | 89757 | 1012 | 5706 | 144 | 143 | 144 | 11370 |
| 9 | F | 5,19 | 689 | 6 | 9 | 199 | 2003 | 154 | 7515 | 9001 | 4780 | 75761 | 7429 | 5513 | 155 | 154 | 155 | 9690 |
| 9 | F | 5,19 | 711 | 7 | 8 | 224 | 2326 | 169 | 7666 | 8075 | 6848 | 97667 | 7838 | 5340 | 170 | 169 | 170 | 8786 |
| 9 | F | 5,19 | 732 | 7 | 8 | 224 | 1787 | 177 | 7601 | 9044 | 5965 | 78499 | 732 | 5814 | 178 | 177 | 177 | 9776 |
| 9 | F | 5,19 | 840 | 7 | 9 | 199 | 1680 | 201 | 7321 | 8786 | 3962 | 78195 | 948 | 5642 | 202 | 201 | 202 | 9625 |
| 9 | F | 5,19 | 668 | 7 | 8 | 199 | 1120 | 212 | 6718 | 7623 | 2326 | 87323 | 991 | 5599 | 213 | 212 | 213 | 8290 |
| 9 | F | 5,19 | 646 | 7 | 8 | 224 | 1314 | 217 | 7494 | 8549 | 4393 | 105578 | 1012 | 6180 | 218 | 217 | 218 | 9195 |
| 9 | F | 5,19 | 711 | 6 | 8 | 199 | 2132 | 303 | 7644 | 8290 | 5685 | 72414 | 969 | 5513 | 304 | 303 | 303 | 9001 |
| 12 | F | 4,59 | 948 | 6 | 8 | 199 | 1680 | 115 | 5642 | 8118 | 3252 | 56558 | 1766 | 3962 | 115 | 114 | 115 | 9066 |
| 12 | F | 4,59 | 625 | 6 | 9 | 224 | 1550 | 123 | 5663 | 8807 | 2907 | 58490 | 1507 | 4113 | 124 | 123 | 124 | 9432 |
| 12 | F | 4,59 | 603 | 5 | 8 | 199 | 1464 | 125 | 4177 | 8807 | 2240 | 50213 | 1443 | 2713 | 126 | 125 | 126 | 9410 |
| 12 | F | 4,59 | 302 | 5 | 7 | 199 | 560 | 135 | 3381 | 4522 | 1529 | 39453 | 323 | 2821 | 135 | 134 | 135 | 4823 |
| 12 | F | 4,59 | 1314 | 7 | 9 | 498 | 2455 | 138 | 5857 | 7623 | 4242 | 49552 | 4199 | 3402 | 138 | 138 | 138 | 8936 |
| 12 | F | 4,59 | 1055 | 6 | 9 | 224 | 1917 | 140 | 5599 | 7687 | 3811 | 48833 | 1658 | 3682 | 140 | 140 | 140 | 8743 |
| 12 | F | 4,59 | 1227 | 6 | 9 | 224 | 2240 | 164 | 5922 | 7128 | 4242 | 62352 | 5125 | 3682 | 165 | 164 | 165 | 8355 |
| 12 | F | 4,59 | 797 | 7 | 8 | 224 | 1723 | 173 | 5060 | 7084 | 3445 | 60420 | 1744 | 3338 | 174 | 173 | 174 | 7881 |
| 12 | F | 4,59 | 1034 | 7 | 9 | 473 | 1873 | 181 | 6029 | 9044 | 3941 | 70628 | 1357 | 4156 | 182 | 181 | 182 | 10078 |
| 12 | F | 4,59 | 1206 | 6 | 9 | 498 | 1895 | 249 | 5814 | 7644 | 3639 | 56558 | 2024 | 3919 | 249 | 249 | 249 | 8850 |
| 15 | F | 5,38 | 1141 | 6 | 9 | 211 | 3316 | 335 | 9281 | 10164 | 6955 | 35694 | 1163 | 5965 | 336 | 335 | 336 | 11305 |
| 15 | F | 5,38 | 1077 | 7 | 9 | 229 | 2347 | 350 | 7838 | 9152 | 5728 | 47063 | 1141 | 5491 | 350 | 350 | 350 | 10228 |
| 15 | F | 5,38 | 948 | 7 | 9 | 229 | 1744 | 352 | 7451 | 9302 | 4673 | 54202 | 1098 | 5706 | 352 | 352 | 352 | 10250 |
| 15 | F | 5,38 | 840 | 7 | 8 | 211 | 1378 | 375 | 5857 | 9087 | 3015 | 37545 | 1077 | 4479 | 375 | 374 | 375 | 9927 |
| 15 | F | 5,38 | 732 | 7 | 9 | 229 | 1227 | 386 | 6266 | 9238 | 3079 | 48385 | 1141 | 5039 | 386 | 385 | 386 | 9970 |
| 15 | F | 5,38 | 840 | 6 | 9 | 211 | 1572 | 388 | 6826 | 9841 | 3682 | 47064 | 1443 | 5254 | 389 | 388 | 389 | 10681 |
| 15 | F | 5,38 | 948 | 7 | 9 | 211 | 1378 | 396 | 8527 | 9647 | 4457 | 39396 | 1055 | 7149 | 396 | 396 | 396 | 10594 |
| 15 | F | 5,38 | 754 | 7 | 9 | 176 | 1507 | 409 | 8204 | 9755 | 4393 | 41511 | 1120 | 6697 | 409 | 409 | 409 | 10508 |
| 15 | F | 5,38 | 689 | 6 | 8 | 264 | 1141 | 424 | 5276 | 9066 | 2735 | 43890 | 1034 | 4134 | 424 | 424 | 424 | 9755 |
| 15 | F | 5,38 | 926 | 7 | 9 | 246 | 1400 | 438 | 7084 | 9152 | 3639 | 43626 | 1141 | 5685 | 438 | 438 | 438 | 10078 |
| 10 | F | 4,64 | 581 | 6 | 8 | 224 | 1292 | 383 | 4608 | 8613 | 1723 | 48167 | 1357 | 3316 | 383 | 383 | 383 | 9195 |
| 10 | F | 4,64 | 345 | 6 | 8 | 212 | 1012 | 438 | 4371 | 8204 | 1486 | 54857 | 1464 | 3359 | 438 | 437 | 438 | 8549 |
| 10 | F | 4,64 | 560 | 6 | 8 | 224 | 1184 | 470 | 4694 | 8312 | 1873 | 44487 | 1507 | 3510 | 470 | 469 | 470 | 8872 |
| 10 | F | 4,64 | 388 | 6 | 9 | 212 | 1206 | 556 | 5060 | 8699 | 2821 | 40139 | 1184 | 3854 | 557 | 556 | 557 | 9087 |
| 10 | F | 4,64 | 560 | 6 | 8 | 236 | 1227 | 612 | 4888 | 8721 | 2907 | 45156 | 1507 | 3661 | 613 | 612 | 613 | 9281 |
| 10 | F | 4,64 | 345 | 6 | 8 | 224 | 1012 | 618 | 5017 | 9259 | 2347 | 52981 | 323 | 4005 | 619 | 618 | 619 | 9604 |
| 10 | F | 4,64 | 366 | 5 | 7 | 224 | 904 | 623 | 1637 | 5405 | 1098 | 45491 | 1077 | 732 | 624 | 623 | 623 | 5771 |
| 16 | F | 4,77 | 991 | 6 | 9 | 199 | 2261 | 508 | 8097 | 9173 | 5082 | 63136 | 1227 | 5836 | 509 | 508 | 508 | 10164 |
| 16 | F | 4,77 | 1034 | 6 | 8 | 174 | 1292 | 527 | 6848 | 8118 | 2799 | 51533 | 1206 | 5556 | 528 | 527 | 528 | 9152 |
| 16 | F | 4,77 | 861 | 5 | 9 | 199 | 1809 | 571 | 7407 | 8829 | 3941 | 50850 | 1163 | 5599 | 572 | 571 | 572 | 9690 |
| 16 | F | 4,77 | 1249 | 7 | 9 | 274 | 2649 | 579 | 7644 | 8140 | 4716 | 62113 | 2692 | 4996 | 580 | 579 | 580 | 9389 |
| 16 | F | 4,77 | 474 | 6 | 8 | 199 | 1292 | 594 | 5276 | 7795 | 2692 | 63820 | 1249 | 3984 | 595 | 594 | 595 | 8269 |
| 16 | F | 4,77 | 1658 | 8 | 9 | 224 | 6029 | 634 | 9066 | 11628 | 7795 | 53580 | 7989 | 3036 | 634 | 633 | 634 | 13286 |
| 16 | F | 4,77 | 474 | 5 | 8 | 224 | 1292 | 640 | 4113 | 7817 | 2046 | 55287 | 1249 | 2821 | 641 | 640 | 641 | 8290 |
| 16 | F | 4,77 | 1206 | 7 | 9 | 199 | 2519 | 660 | 8463 | 9130 | 5146 | 54605 | 2089 | 5943 | 661 | 660 | 661 | 10336 |
| 16 | F | 4,77 | 668 | 6 | 8 | 199 | 1271 | 671 | 5319 | 7881 | 2541 | 72351 | 1163 | 4048 | 672 | 671 | 672 | 8549 |
| 16 | F | 4,77 | 517 | 5 | 8 | 224 | 1292 | 687 | 6460 | 8312 | 3144 | 63478 | 1163 | 5168 | 687 | 687 | 687 | 8829 |
| 11 | F | 4,47 | 495 | 5 | 8 | 176 | 861 | 54 | 4242 | 8376 | 1895 | 70628 | 883 | 3381 | 55 | 54 | 54 | 8872 |
| 11 | F | 4,47 | 474 | 5 | 8 | 211 | 581 | 59 | 3165 | 7817 | 1464 | 52578 | 560 | 2584 | 59 | 59 | 59 | 8290 |
| 11 | F | 4,47 | 797 | 7 | 8 | 176 | 1206 | 112 | 7774 | 9066 | 4544 | 67881 | 840 | 6568 | 113 | 112 | 113 | 9862 |
| 11 | F | 4,47 | 991 | 7 | 9 | 176 | 2885 | 119 | 7257 | 8484 | 6029 | 77690 | 1249 | 4371 | 120 | 119 | 119 | 9475 |
| 11 | F | 4,47 | 818 | 5 | 8 | 176 | 926 | 125 | 6029 | 8269 | 2778 | 60426 | 926 | 5103 | 126 | 125 | 126 | 9087 |
| 11 | F | 4,47 | 517 | 5 | 8 | 194 | 1206 | 268 | 8161 | 8979 | 5383 | 68274 | 517 | 6955 | 268 | 267 | 268 | 9496 |
| 11 | F | 4,47 | 538 | 5 | 9 | 176 | 1227 | 289 | 7386 | 8463 | 3790 | 75729 | 818 | 6159 | 290 | 288 | 289 | 9001 |
| 11 | F | 4,47 | 603 | 5 | 8 | 176 | 969 | 340 | 3919 | 7278 | 2089 | 60818 | 926 | 2950 | 340 | 339 | 340 | 7881 |
| 11 | F | 4,47 | 517 | 6 | 8 | 194 | 668 | 345 | 5060 | 7924 | 1766 | 54932 | 625 | 4393 | 346 | 345 | 346 | 8441 |
| 11 | F | 4,47 | 603 | 6 | 8 | 194 | 861 | 372 | 4630 | 7644 | 1938 | 58464 | 861 | 3768 | 373 | 372 | 373 | 8247 |
| 1 | F | 4,19 | 818 | 7 | 9 | 211 | 3036 | 195 | 8484 | 9862 | 6891 | 50402 | 8807 | 5448 | 195 | 194 | 195 | 10681 |
| 1 | F | 4,19 | 754 | 6 | 9 | 212 | 3295 | 197 | 8592 | 9862 | 6826 | 81602 | 1077 | 5297 | 198 | 197 | 198 | 10616 |
| 1 | F | 4,19 | 689 | 6 | 9 | 199 | 1787 | 201 | 5534 | 8829 | 3941 | 76148 | 1012 | 3747 | 202 | 201 | 202 | 9518 |
| 1 | F | 4,19 | 474 | 5 | 7 | 282 | 797 | 209 | 1421 | 7235 | 991 | 45165 | 797 | 625 | 210 | 209 | 210 | 7709 |
| 1 | F | 4,19 | 732 | 8 | 9 | 187 | 1615 | 241 | 7128 | 9259 | 4134 | 50838 | 1357 | 5513 | 242 | 241 | 242 | 9991 |
| 1 | F | 4,19 | 861 | 6 | 8 | 199 | 1464 | 245 | 4565 | 7774 | 3122 | 38619 | 1184 | 3101 | 245 | 244 | 245 | 8635 |
| 1 | F | 4,19 | 689 | 6 | 9 | 199 | 2993 | 251 | 8183 | 8829 | 5556 | 43201 | 7580 | 5190 | 251 | 250 | 251 | 9518 |
| 1 | F | 4,19 | 603 | 6 | 8 | 224 | 1055 | 256 | 4802 | 8441 | 1960 | 29892 | 668 | 3747 | 256 | 256 | 256 | 9044 |
| 1 | F | 4,19 | 452 | 5 | 8 | 199 | 1034 | 287 | 4091 | 8290 | 2067 | 45383 | 1335 | 3058 | 287 | 287 | 287 | 8743 |
| 1 | F | 4,19 | 517 | 5 | 8 | 176 | 904 | 289 | 3467 | 8829 | 1292 | 38183 | 603 | 2563 | 290 | 289 | 290 | 9345 |
| 17 | M | 4,41 | 560 | 7 | 8 | 199 | 1055 | 184 | 7752 | 8699 | 4630 | 75984 | 7644 | 6697 | 185 | 183 | 184 | 9259 |
| 17 | M | 4,41 | 581 | 7 | 8 | 199 | 1077 | 193 | 7666 | 8441 | 4285 | 78048 | 969 | 6589 | 194 | 193 | 194 | 9022 |
| 17 | M | 4,41 | 581 | 7 | 8 | 199 | 1055 | 240 | 7515 | 8333 | 4113 | 86720 | 711 | 6460 | 241 | 240 | 241 | 8915 |
| 17 | M | 4,41 | 581 | 7 | 8 | 187 | 1120 | 244 | 6934 | 7795 | 3424 | 73092 | 1206 | 5814 | 244 | 243 | 244 | 8376 |
| 17 | M | 4,41 | 560 | 5 | 8 | 199 | 797 | 251 | 6417 | 8097 | 2433 | 61942 | 1034 | 5620 | 251 | 250 | 251 | 8656 |
| 17 | M | 4,41 | 517 | 6 | 8 | 187 | 948 | 270 | 5900 | 7881 | 1550 | 44185 | 711 | 4953 | 271 | 270 | 270 | 8398 |
| 17 | M | 4,41 | 388 | 6 | 8 | 249 | 1034 | 282 | 5276 | 8204 | 2304 | 91675 | 1141 | 4242 | 283 | 282 | 283 | 8592 |
| 17 | M | 4,41 | 603 | 7 | 8 | 187 | 1486 | 406 | 7709 | 8226 | 6288 | 61117 | 8075 | 6223 | 406 | 406 | 406 | 8829 |
| 17 | M | 4,41 | 345 | 6 | 8 | 199 | 1012 | 421 | 7257 | 8721 | 2907 | 73505 | 1055 | 6245 | 422 | 420 | 422 | 9066 |
| 17 | M | 4,41 | 345 | 6 | 8 | 199 | 991 | 433 | 7106 | 8527 | 3015 | 92089 | 1034 | 6115 | 434 | 433 | 434 | 8872 |
| 18 | M | 4,98 | 2046 | 6 | 9 | 194 | 3618 | 163 | 8118 | 8764 | 6826 | 47523 | 7278 | 4500 | 164 | 163 | 164 | 10810 |
| 18 | M | 4,98 | 1723 | 6 | 9 | 158 | 5039 | 174 | 8118 | 9109 | 7300 | 55909 | 7472 | 3079 | 174 | 173 | 174 | 10831 |
| 18 | M | 4,98 | 1034 | 6 | 8 | 194 | 1594 | 202 | 4759 | 8010 | 2563 | 61900 | 1873 | 3165 | 203 | 202 | 202 | 9044 |
| 18 | M | 4,98 | 1055 | 4 | 9 | 220 | 3962 | 238 | 8032 | 9432 | 5620 | 39136 | 1034 | 4070 | 239 | 238 | 239 | 10487 |
| 18 | M | 4,98 | 1249 | 6 | 8 | 229 | 2390 | 241 | 5771 | 7321 | 3036 | 50318 | 2584 | 3381 | 241 | 240 | 241 | 8570 |
| 18 | M | 4,98 | 1034 | 6 | 9 | 176 | 3015 | 387 | 7795 | 9841 | 6589 | 53114 | 6826 | 4780 | 388 | 387 | 388 | 10874 |
| 18 | M | 4,98 | 1206 | 5 | 9 | 176 | 2864 | 390 | 7386 | 8161 | 4371 | 56708 | 2756 | 4522 | 390 | 389 | 390 | 9367 |
| 18 | M | 4,98 | 991 | 5 | 9 | 387 | 2993 | 394 | 8226 | 8786 | 5190 | 52315 | 1012 | 5233 | 395 | 394 | 395 | 9776 |
| 18 | M | 4,98 | 1249 | 6 | 8 | 174 | 2735 | 396 | 5556 | 7171 | 2972 | 47124 | 2842 | 2821 | 397 | 396 | 397 | 8420 |
| 18 | M | 4,98 | 1271 | 6 | 9 | 194 | 2799 | 466 | 7278 | 7881 | 4307 | 59504 | 2864 | 4479 | 466 | 465 | 466 | 9152 |
| 19 | M | 4,74 | 452 | 6 | 8 | 249 | 991 | 206 | 4780 | 8053 | 1658 | 52209 | 1227 | 3790 | 207 | 206 | 207 | 8506 |
| 19 | M | 4,74 | 495 | 6 | 8 | 224 | 1271 | 252 | 6697 | 8161 | 2476 | 50174 | 1314 | 5426 | 252 | 251 | 252 | 8656 |
| 19 | M | 4,74 | 452 | 4 | 7 | 236 | 603 | 266 | 1507 | 6395 | 1034 | 47802 | 991 | 904 | 267 | 266 | 266 | 6848 |
| 19 | M | 4,74 | 474 | 6 | 8 | 194 | 1034 | 278 | 2885 | 6891 | 1507 | 52887 | 1314 | 1852 | 279 | 278 | 279 | 7364 |
| 19 | M | 4,74 | 560 | 7 | 9 | 211 | 1594 | 287 | 7558 | 8463 | 4673 | 62718 | 1335 | 5965 | 288 | 287 | 288 | 9022 |
| 19 | M | 4,74 | 538 | 7 | 9 | 176 | 1357 | 290 | 7321 | 8635 | 3316 | 52887 | 1615 | 5965 | 290 | 289 | 290 | 9173 |
| 19 | M | 4,74 | 517 | 6 | 8 | 194 | 926 | 328 | 4350 | 7580 | 1658 | 44750 | 1249 | 3424 | 328 | 327 | 328 | 8097 |
| 19 | M | 4,74 | 861 | 7 | 9 | 220 | 2218 | 342 | 7989 | 8872 | 5362 | 45768 | 2864 | 5771 | 342 | 341 | 342 | 9733 |
| 19 | M | 4,74 | 452 | 6 | 8 | 220 | 948 | 348 | 6417 | 8312 | 1787 | 53565 | 538 | 5469 | 349 | 348 | 349 | 8764 |
| 19 | M | 4,74 | 452 | 6 | 8 | 185 | 1206 | 371 | 3575 | 7321 | 1658 | 43395 | 1594 | 2369 | 372 | 371 | 372 | 7774 |
| 20 | M | 4,13 | 603 | 7 | 9 | 199 | 1723 | 119 | 8376 | 9712 | 6288 | 53485 | 1314 | 6654 | 120 | 119 | 120 | 10314 |
| 20 | M | 4,13 | 345 | 6 | 8 | 162 | 991 | 125 | 4307 | 8850 | 1335 | 60313 | 1335 | 3316 | 125 | 124 | 125 | 9195 |
| 20 | M | 4,13 | 646 | 5 | 9 | 187 | 1206 | 134 | 8183 | 9410 | 5836 | 54623 | 1077 | 6977 | 135 | 134 | 135 | 10056 |
| 20 | M | 4,13 | 366 | 6 | 9 | 174 | 1314 | 140 | 7903 | 9539 | 4285 | 74727 | 1400 | 6589 | 141 | 140 | 141 | 9905 |
| 20 | M | 4,13 | 560 | 6 | 8 | 176 | 991 | 160 | 4996 | 8699 | 1357 | 74727 | 1292 | 4005 | 161 | 160 | 161 | 9259 |
| 20 | M | 4,13 | 517 | 6 | 8 | 187 | 1077 | 170 | 7343 | 9022 | 3036 | 73969 | 1271 | 6266 | 171 | 170 | 171 | 9539 |
| 20 | M | 4,13 | 861 | 5 | 8 | 185 | 1335 | 236 | 8398 | 9281 | 6718 | 70935 | 1249 | 7063 | 237 | 236 | 236 | 10142 |
| 20 | M | 4,13 | 560 | 6 | 8 | 212 | 1098 | 250 | 7946 | 9259 | 3747 | 108488 | 969 | 6848 | 252 | 250 | 251 | 9819 |
| 20 | M | 4,13 | 861 | 7 | 9 | 187 | 3079 | 273 | 8247 | 9152 | 6783 | 76624 | 969 | 5168 | 273 | 272 | 273 | 10013 |
| 20 | M | 4,13 | 904 | 6 | 8 | 199 | 1378 | 315 | 8613 | 9281 | 6934 | 80796 | 1314 | 7235 | 316 | 315 | 316 | 10185 |
| 21 | M | 4,68 | 560 | 6 | 8 | 187 | 1292 | 164 | 7580 | 9281 | 6331 | 47754 | 1271 | 6288 | 165 | 164 | 165 | 9841 |
| 21 | M | 4,68 | 560 | 5 | 8 | 212 | 1292 | 167 | 6934 | 8204 | 3144 | 54183 | 1292 | 5642 | 167 | 166 | 167 | 8764 |
| 21 | M | 4,68 | 517 | 7 | 9 | 174 | 1981 | 191 | 7171 | 8807 | 5836 | 44081 | 6266 | 5190 | 191 | 191 | 191 | 9324 |
| 21 | M | 4,68 | 732 | 5 | 8 | 212 | 1378 | 199 | 7149 | 7946 | 5103 | 52805 | 991 | 5771 | 200 | 199 | 199 | 8678 |
| 21 | M | 4,68 | 280 | 6 | 8 | 199 | 560 | 261 | 6783 | 9195 | 1421 | 57146 | 302 | 6223 | 262 | 261 | 262 | 9475 |
| 21 | M | 4,68 | 926 | 6 | 8 | 187 | 1507 | 272 | 7020 | 8463 | 3639 | 57398 | 1443 | 5513 | 273 | 272 | 273 | 9389 |
| 21 | M | 4,68 | 280 | 7 | 8 | 187 | 1077 | 295 | 7494 | 8613 | 5362 | 55197 | 1012 | 6417 | 296 | 295 | 296 | 8893 |
| 21 | M | 4,68 | 560 | 5 | 8 | 224 | 1120 | 326 | 5857 | 7558 | 1529 | 57397 | 1400 | 4737 | 327 | 326 | 327 | 8118 |
| 21 | M | 4,68 | 495 | 6 | 8 | 199 | 1163 | 328 | 5469 | 7752 | 1507 | 36275 | 1206 | 4307 | 328 | 328 | 328 | 8247 |
| 21 | M | 4,68 | 1098 | 5 | 8 | 212 | 5211 | 355 | 8097 | 8699 | 7149 | 47296 | 1314 | 2885 | 356 | 355 | 355 | 9798 |
| 22 | M | 3,85 | 1335 | 7 | 9 | 373 | 2175 | 236 | 8161 | 10530 | 4522 | 70968 | 2089 | 5986 | 237 | 236 | 236 | 11865 |
| 22 | M | 3,85 | 517 | 6 | 8 | 187 | 1314 | 260 | 5276 | 9970 | 3381 | 66601 | 1378 | 3962 | 260 | 259 | 260 | 10487 |
| 22 | M | 3,85 | 840 | 7 | 9 | 174 | 1507 | 262 | 4845 | 9324 | 2369 | 65509 | 1637 | 3338 | 263 | 262 | 262 | 10164 |
| 22 | M | 3,85 | 517 | 5 | 8 | 187 | 991 | 266 | 4544 | 8678 | 2649 | 68785 | 625 | 3553 | 267 | 266 | 267 | 9195 |
| 22 | M | 3,85 | 646 | 6 | 9 | 249 | 2864 | 295 | 8140 | 10465 | 4651 | 66055 | 2692 | 5276 | 296 | 295 | 295 | 11111 |
| 22 | M | 3,85 | 840 | 5 | 9 | 187 | 2003 | 301 | 7364 | 8786 | 5125 | 69331 | 1464 | 5362 | 302 | 301 | 302 | 9625 |
| 22 | M | 3,85 | 1357 | 6 | 9 | 784 | 2627 | 334 | 6977 | 8420 | 5211 | 76972 | 5879 | 4350 | 335 | 333 | 334 | 9776 |
| 22 | M | 3,85 | 1335 | 7 | 9 | 460 | 2024 | 337 | 7149 | 9238 | 4651 | 104814 | 1594 | 5125 | 338 | 337 | 338 | 10573 |
| 22 | M | 3,85 | 1550 | 8 | 9 | 597 | 2584 | 349 | 7838 | 9776 | 5190 | 63872 | 2541 | 5254 | 350 | 349 | 350 | 11327 |
| 22 | M | 3,85 | 1615 | 8 | 9 | 448 | 4307 | 450 | 8678 | 10358 | 6611 | 81886 | 8592 | 4371 | 451 | 449 | 450 | 11973 |

Measured data from acoustical analysis.

(ID) Identity corresponding to DFA results. (Sex) Sex of tested individual. (Weight) Body weight. (F5) Frequency 5%. (MinE) Minimum Entropy. (AggE) Aggregate Entropy. (LowF) Low frequency. (Q1F) First quartile frequency. (Q1Time) First quartile time. (Q3F) Third quartile frequency. (BW90) Bandwidth 90%. (CentF) Center frequency. (Call dur) Sample Length. (PeakF) Peak frequency. (IQR) Inter- quartile range. (T95) Time 95%. (T5) Time 5%. (Q3T) Third quartile time. (F95) Frequency 95%.
